# An adjustment framework for mitigating the impact of rail transit construction on bus operations

**DOI:** 10.1038/s41598-022-14543-w

**Published:** 2022-06-20

**Authors:** Qinhe An, Jie Ma, Jingxu Chen, Wenhao Li, Yiran Wang

**Affiliations:** 1 School of Transportation, Southeast University, Nanjing, China; 2 Jiangsu Key Laboratory of Urban Intelligent Transportation Systems, Southeast University, Nanjing, China

**Keywords:** Civil engineering, Software

## Abstract

During the construction of rail transit, some road resources are occupied under construction and hence the capacity of affected road sections is reduced. Due to such temporary impact, the low operational efficiency of bus routes when passing these road sections is following suit, which is inevitably detrimental to the short-term traffic conditions and the urban environment. This paper proposes a three-step adjustment framework to actively mitigate the impact of rail transit construction on bus operations. In Step I, a criterion is developed for selecting bus routes significantly affected due to rail transit construction. In Step II, we design a stop-skipping strategy and truncation strategy, and check whether they are opportune to ameliorate the service of each affected bus route. Yet, these two strategies result in a loss of ridership since some previous stops are not served. In Step III, the feeder bus service is further designed to guarantee spatial coverage nearby the construction area. A mixed-integer linear programming model is developed to determine the optimal configuration of the feeder bus service incorporating two strategies. Finally, a case study based on the Sioux-Falls network is carried out to demonstrate that the proposed adjustment framework is conducive to mitigating negative construction effects and reducing costs.

## Introduction

Targeting the sustainable development of urban transport systems, public transit is commonly regarded as an effective way to alleviate traffic congestion, reduce emissions, and address other environmental issues resulting from transportation^1,2^. In the process of continuous urbanization, the urban population and associated travel demand keep growing. Many metropolitan areas attempt to extend their service coverage of public transit by constructing new rail transit lines to accommodate the increasing travel demand^3^. Rail transit lines are generally built along transportation corridors with high passenger flow. The passenger flow of these corridors is previously serviced by bus transit.

During the construction of rail transit, the capacity of road sections under construction is reduced. The construction period of rail transit is long, which inevitably affects the operational efficiency and level of service of related bus routes. Some passengers who originally took buses may choose other modes of transport, such as private cars. This is not conducive to mitigating traffic congestion and the sustainable development of the city. Thus, some active adjustment measures need to be taken to improve the inefficient bus operations resulting from rail transit construction.

The bulk of the current literature focuses on the issue of coordination between rail transit and buses after the completion of rail transit^4–7^. Few studies pay attention to the impact on bus transit during the construction of rail transit. After the completion of rail transit, the purpose of bus route adjustment is to reduce duplication with rail transit lines and well couple with rail transit in service. While during the construction of rail transit, bus routes adjacent to the construction area may be influenced. These bus routes should meet basic demand and meantime maintain certain service quality via reducing the impact of construction on bus operations. Hence, an adjustment framework for mitigating the impact of rail transit construction on bus operations is necessary, which is addressed in this paper.

### Literature review

After the completion of rail transit, the bus routes should be adjusted to coordinate with the in-use rail transit lines. Many researchers have made efforts to the coordination problem between bus and rail transit. A multi-objective model was developed for designing an integrated rail transit and bus network on a transit corridor with a new rail transit route^8^. Bi-level programming model was also proposed to optimize the adjustment schemes of bus routes, taking the impacts of the urban rail transit network into account to minimize the passenger travel cost and maximize the benefits of bus operators^5^. The reconfiguration of bus services in an urban area with a newly constructed rail transit system was investigated; the rail transit services are taken as the backbone, and the bus services are reconfigured to better integrate with rail services^9^. Recently, Cui et al. proposed a model for the adjustment of bus routes along a rail transit line; the generalized travel time costs and travel time savings proportion in the collinear section of rail transit and bus was optimized^6^. Wei et al. studied how to adjust and optimize the original bus routes based on bus and metro integration^7^.

During rail construction or road maintenance, several work zones are formed, which occupy the road resource and influence the operation of ground transportation^10^. Several studies have been conducted to analyze the impact of work zones on ground transportation. The optimization of project schedule and traffic flow organization was investigated to reduce traffic delay and travel costs resulting from the disturbance of work zones^11–13^. Concerning the impact of rail construction on bus routes, Huang et al. proposed a method to evaluate the performances before and after the adjustment of the bus routes^14^.

As an important complement, the feeder bus service effectively extends the service scope of rail transit and solves the problem of short-distance travel^15^. Verma et al. discussed a model for developing optimal feeder bus routes, within an integrated mass transit planning framework, for urban rail transit stations^16^. Xiong et al. constructed a route design model for feeder buses to minimize the total cost and obtained the optimal stations using heuristics algorithms^17^. Lin and Wong presented a feeder bus route design model, to minimize route length and travel time and maximize service coverage; a multi-objective programming approach was developed to solve the model^18^. The optimized results dominate the existing bus routes in terms of all objectives and stakeholder concerns, except for the risk of trip generation estimation that bus service providers are mostly concerned with. Zheng et al. proposed a nonlinear mixed-integer programming model to address multiple circular feeder bus route design problems^19^. It found that in areas with a mature public transport system, combining adjusting existing bus routes and designing new feeder routes together is a more cost-effective way. Recently, Cao et al. studied a feeder bus route optimization problem to minimize the sum of bus operation cost and passenger travel cost^20^. The results show that compared with the existing transit network, the optimal scheme improved the passenger-carrying rate, reducing the per capita travel cost and improving the overall operating efficiency. These studies have reported many positive findings on the operations of feeder buses for in-use transit systems. However, the role of feeder buses during the construction period of rail transit has not been studied particularly.

The stop-skipping strategy has been studied by many researchers to increase bus operating speeds^3,21–24^. Under the stop-skipping service pattern, bus trips skip some of the stops of the original bus route^25^. Except for designing stop-skipping routes in tactical planning, the stop-skipping strategy can also be used for real-time and emergency bus management. Zhang et al. developed a real-time agent-based simulation model to optimize the dynamic bus stop-skipping and holding schemes^26^. The objective was to reduce passenger waiting and on-board time and improve the reliability of bus service. Zhao et al. used a stop-skipping method for bus fleet scheduling to meet sudden increases in demand^27^. The stop-skipping decision and fleet size are modeled as a dynamic program to obtain the optimal strategy that minimizes the passenger waiting time. The proposed approach reduced the total waiting time for passengers by more than 20%.

In conclusion, the impact of rail transit construction on bus operations and the adjustment of bus services from the network level have not been studied particularly, though some existing studies provide a reference for the bus operations during the rail construction. In addition, to the best of our knowledge, there is no research on the application of feeder bus service to the bus adjustment during the construction period of rail transit.

### Objectives and contributions

The primary objective of this study is to propose an adjustment framework in an effort to actively mitigate the impact of rail transit construction on bus operations. For bus routes that need to be adjusted, we first check whether the stop-skipping and truncation strategies are effective in reducing the total cost. Bus routes where these two strategies are invalid are optimized through the design of the feeder bus route. A mixed-integer linear programming model (MILP) is developed to determine the optimal configuration of the feeder bus service. To sum up, the contributions of this paper are threefold: (1) A three-step adjustment framework is proposed to actively mitigate the impact of rail transit construction on bus operations. (2) We propose a quantitative indicator to determine whether the stop-skipping and truncating strategies are effective. (3) The feeder bus route is designed to optimize the bus routes affected by the construction of rail transit. Affected bus routes bypassing the construction sections directly would affect the ride of some passengers, the feeder bus service can address this issue. The proposed adjustment framework could reduce total system cost and relieve the impact of rail transit construction on passengers, which is conducive to mitigating traffic congestion and enhancing the sustainable development of the city.

The remainder of this paper is organized as follows: In “Problem description”, we formally describe the problem. In “Three-step adjustment framework”, we explain the implementation of the three-step adjustment framework and formulate the problem. “Case study” presents the results from our numerical experiments. Finally, in “Conclusions”, we provide some concluding remarks and directions for future research.

## Problem description

Consider a rail transit line under construction in the urban transportation network. Some road resources are occupied during the construction and hence the capacity of affected road sections is reduced. As a result, the low operational efficiency and poor service level of related bus routes when passing these road sections are following suit. Let $L$ represent the set of bus routes affected by construction. Hereinafter, the affected bus routes refer to bus routes partially collinear with the road sections under construction.

For ease of presentation, the notations for parameters and variables used in this paper are summarized in Table 1.

**Table 1:** Summary of the notations used in this paper (in alphabetical order).

Sets	
$A_{l}$	The affected section of bus route $l$
$D_{l}$	The downstream section of the affected section for bus route $l$
$L$	Set of bus routes affected by construction
$\overline{L}$	Set of bus routes affected by construction significantly and need adjusting
$L_{1}$	Set of bus routes optimized by stop-skipping strategy
$L_{2}$	Set of bus routes optimized by truncation strategy
$L_{3}$	Set of bus routes optimized by feeder bus service
$U_{l}$	The upstream section of the affected section of bus route $l$

Parameters	
$c_{0}$	Constant parameter for the unit operating cost per vehicle-hour
$Cap$	Constant parameter for the capacity of a bus
$d_{i,l}$	Dwell time at stop $i$ for bus route $l$
$f$	The frequency of feeder bus service
$LF_{\max }$	The maximum load factor of the feeder bus service
$q_{rs,l}$	Traffic demand from bus stop $r$ to $s$ on bus route $l$
$q_{rs,l}^{skip}$	Traffic demand from bus stop $r$ to $s$ on bus route $l$ after implementing the stop-skipping strategy
$q_{rs,l}^{tru}$	Traffic demand from bus stop $r$ to $s$ on bus route $l$ after implementing the truncation strategy
$R_{l}$	An indicator for judging whether bus route $l$ needs to be adjusted
$R_{0}$	The critical value of indicator $R_{l}$, bus route $l$ needs to be adjusted if $R_{l} > R_{0}$
$t_{a}$	Constant parameter for the average alighting time of one passenger
$t_{b}$	Constant parameter for the average boarding time of one passenger
$t_{ij}$	Bus travel time of link $(i,j)$ on bus route $l$ before construction
$t_{ij}^{c}$	Bus travel time of link $(i,j)$ on bus route $l$ during construction
$T_{l}$	Roundtrip time of bus route $l$ before construction
$T_{l}^{c}$	Roundtrip time of bus route $l$ during construction
$T_{l}^{s}$	Roundtrip time of bus route $l$ during construction if the stop-skipping strategy is implemented
$T_{l}^{T}$	Roundtrip time of bus route $l$ during construction if the truncation strategy is implemented
$v_{ij,l}$	Passenger flow on link $(i,j)$ of bus route $l$
$\gamma$	The penalty for losing one passenger
$\lambda$	Passenger’s value of time

Decision variables	
$x_{ij}$	If the feeder bus service passes through node $j$ immediately after node $i$, then $x_{ij} = 1$; otherwise $x_{ij} = 0$
$v_{ij}$	Passenger flow on link $(i,j)$ of feeder bus service

**Figure 1:**
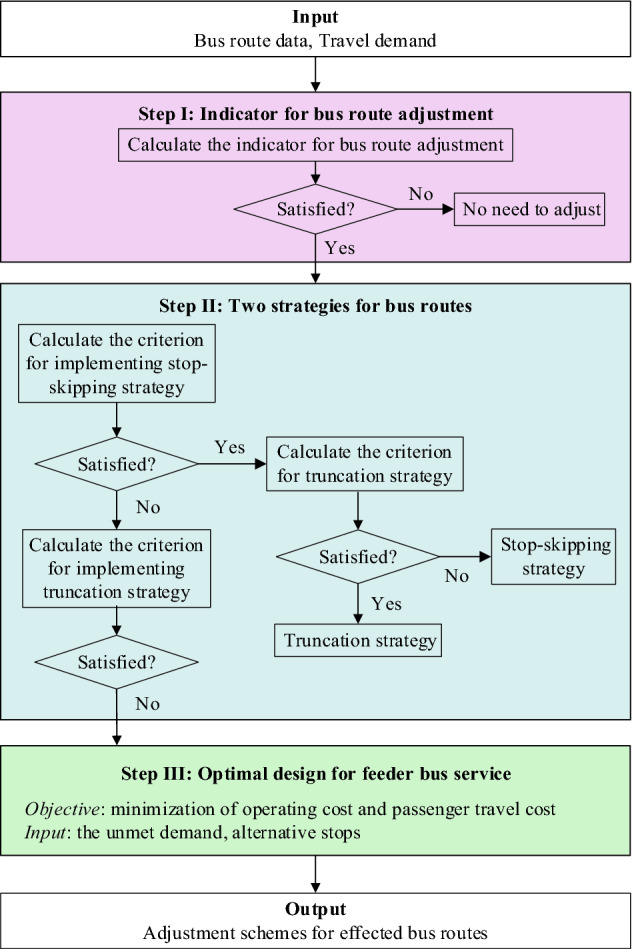
Flowchart for bus adjustment framework.

A three-step adjustment framework is proposed to actively mitigate the impact of rail transit construction on bus operations and its flowchart is shown in Fig. 1.

Each bus route is affected by construction to a different degree. In Step I, an indicator is first proposed to evaluate whether the bus route is significantly affected due to rail transit construction and needs to be adjusted. The set of bus routes that need to be adjusted is denoted by $\overline{L}$.

**Figure 2:**
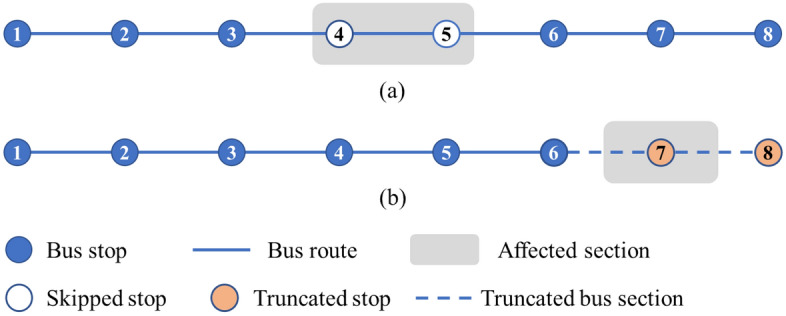
Illustration of the two strategies: (**a**) stop-skipping strategy and (**b**) truncation strategy.

In Step II, we design two strategies, i.e., the stop-skipping strategy and truncation strategy, and check whether they are opportune to ameliorate the service of each bus route in $\overline{L}$. Every bus route is divided into three sections, including the affected section (collinear with rail transit line under construction) and its upstream and downstream sections, which are denoted by $A_{l}$, $U_{l}$, and $D_{l}$, respectively. In Fig. 2a, the affected section, and its upstream and downstream sections of bus route 1 are $A_{1} = \left\{ {4,5} \right\}$, $U_{1} = \left\{ {1,2,3} \right\}$, and $D_{1} = \left\{ {6,7,8} \right\}$, respectively. The stop-skipping strategy in this paper refers to skipping the bus stops in the affected section, for example, bus stops 4 and 5 are skipped in Fig. 2a. The truncation strategy refers to skipping the bus stops and sections both in the affected section and the downstream section. In Fig. 2b, the bus route skips bus stops 7 and 8, as well as bus sections between stop 6 and stop 8. Then the terminal stop of the route changes from stop 8 to stop 6.

These two strategies may reduce operating costs, as well as some passengers’ travel costs because they experience a shorter travel time. Yet, the two strategies may result in a loss of ridership since some stops are not served. Passengers who start or end at these stops will not be able to ride. Assume the penalty for losing one passenger is $\gamma$. The sum of operating costs and passenger travel costs before and after the implementation of the strategies are calculated to determine whether taking the strategy, i.e., if a strategy can reduce the cost of one bus route, then it is implemented to adjust the bus route. Specifically, if a bus route meets the criteria of the stop-skipping and the truncation strategies at the same time, then the truncation strategy is adopted. This is because the truncation strategy is more conducive to mitigating traffic congestion, thereby reducing the impact of construction on traffic conditions and the urban environment. Besides, compared to the stop-skipping strategy, the truncation strategy is less confused for passengers and thus simpler to implement. In Step III, the feeder bus service is further designed to guarantee spatial coverage nearby the construction area. A mixed-integer linear programming model is developed to determine the optimal configuration of the feeder bus service incorporating two strategies.

## Three-step adjustment framework

### Step I: adjustment indicator

As the rail transit is under construction, the affected bus routes need to serve their original passenger flow and cannot bypass the construction section directly. Whether an affected bus route needs to be adjusted is based on the impact degree of construction on the bus route. Travel time is found to be one of the most important factors in travel choices^28^. Besides, bus travel speed (which can be reflected in travel time) is a fundamental indicator for the bus service evaluation^29^. Therefore, we choose the increasing rate of roundtrip time as the indicator for selecting bus routes which are significantly affected due to rail transit construction and necessitate being adjusted. The increasing rate of roundtrip time of bus route $l \in L$ is calculated as follows:

1 $$R_{l} = \frac{{T_{l}^{c} - T_{l} }}{{T_{l} }} \times 100\% ,$$

where $T_{l}$ and $T_{l}^{c}$ are the roundtrip time of bus route $l \in L$ before and during construction. We future define a critical value $R_{0}$ for indicator $R_{l}$ to judge whether bus route $l$ needs adjusting, that is, if $R_{l} > R_{0}$, bus route $l \in L$ needs adjusting.

The set of bus routes that satisfy $R_{l} > R_{0}$ indicates that these routes are significantly affected by rail transit construction, which is denoted by $\overline{L}$.

### Step II: two strategies

#### Stop-skipping strategy

Route cost is defined as the sum of the operating cost and passenger travel cost of a bus route. For any bus route, if the stop-skipping strategy can reduce the route cost, then the stop-skipping strategy is adopted. Hereinafter, all the costs calculated refer to the hourly costs. Assume the construction only affects link travel time and does not affect dwell time at bus stops. The total travel time of passengers on bus route $l \in L$ under construction is as follows:

2 $$T_{0,l} = \sum\limits_{(i,j)} {\left[ {v_{ij,l} \cdot (t_{ij}^{c} + d_{i,l} )} \right]} , \, (i,j) \in A_{l} \cup U_{l} \cup D_{l} ,$$

where $v_{ij,l}$ is the flow of link $(i,j)$ on bus route $l \in L$; $t_{ij}^{c}$ is the bus travel time of link $(i,j)$ on bus route $l$ during construction; $d_{i,l}$ is dwell time at stop $i$ for bus route $l$. $v_{ij,l}$ is given by:

3 $$v_{ij,l} = \sum\limits_{r \le i} {\sum\limits_{s \ge j} {q_{rs,l} } } .$$

It is assumed that dwell time only depends on the number of boarding and alighting passengers, and the effects of other factors are ignored. The bus vehicles in most cities have two doors, one for boarding and one for alighting, thus at each stop, the dwell time is equal to the maximum value between boarding time and alight time^23^:

4 $$d_{i,l} = \max (t_{a} \cdot v_{i,a} ,t_{b} \cdot v_{i,b} ),$$

where $t_{a}$ and $t_{b}$ are constant parameters for the average alighting/boarding time of one passenger. $v_{i,a}$ and $v_{i,b}$ are the number of passengers alight/board at bus stop $i$, which are given by

5 $$v_{i,a} = \sum\limits_{r \le i} {q_{rs,l} } ,$$

6 $$v_{i,b} = \sum\limits_{s \ge i} {q_{rs,l} } .$$

The total operating cost of bus route $l$ under construction is as follows:

7 $$OC_{0} = c_{0} \cdot T_{l}^{c} ,$$

where $c_{0}$ is a constant parameter for the unit operating cost per vehicle-hour; $T_{l}^{c}$ is the roundtrip time of bus route $l \in L$ during construction, which is given by

8 $$T_{l}^{c} = \sum\limits_{(i,j)} {(t_{ij}^{c} + d_{i} )} , \, (i,j) \in A_{l} \cup U_{l} \cup D_{l} .$$

With the stop-skipping strategy, the total travel time of passengers on bus route $l \in L$ under construction is as follows:

9 $$T_{1,l} = \sum\limits_{{(i,j) \in A_{l} }} {\left[ {v_{ij,l}^{s} \cdot (t_{ij}^{c} )} \right]} + \sum\limits_{{(i,j) \in D_{l} }} {[v_{ij,l}^{s} \cdot (t_{ij}^{c} + d_{i,l} )]} ,$$

where $v_{ij,l}^{s}$ is the flow of link $(i,j)$ on bus route $l \in L$ after the implementation of the stop-skipping strategy and is given by

10 $$v_{ij,l}^{s} = \sum\limits_{r \le i} {\sum\limits_{s \ge j} {q_{rs,l}^{skip} } } ,$$

where $q_{rs,l}^{skip}$ is the traffic demand from bus stop $r$ to $s$ on bus route $l \in L$ after implementing the stop-skipping strategy and is given by

11 $$q_{rs,l}^{skip} = \left\{ {\begin{array}{*{20}c} {0,} & {if \, r/s \in A_{l} } \\ {q_{rs,l} ,} & {else.} \\ \end{array} } \right.$$

With the stop-skipping strategy, the total operating cost of bus route $l \in L$ is as follows:

12 $$OC_{1,l} = c_{0} \cdot T_{l}^{s} ,$$

where $T_{l}^{s}$ is the roundtrip time of bus route $l \in L$ during construction after implementing the stop-skipping strategy, which is calculated as

13 $$T_{l}^{s} = \sum\limits_{{(i,j) \in A_{l} }} {t_{ij}^{c} } + \sum\limits_{{(i,j) \in U_{l} \cup D_{l} }} {(t_{ij}^{c} + d_{i} )} .$$

The passenger loss caused by the stop-skipping strategy is:

14 $$P_{1,l} = \gamma \cdot \sum\limits_{r} {\sum\limits_{s} {q_{rs,l} } , \, r/s \in A_{l} } .$$

The criterion for implementing the stop-skipping strategy $c_{1}$ is given by:

15 $$(\lambda \cdot T_{1,l} + OC_{1,l} + P_{1,l} ) - (\lambda \cdot T_{0,l} + OC_{0,l} ) < 0,$$

where $\lambda$ is passengers’ value of time. The two terms on the left-hand side of inequation (15) represent the route cost with and without the stop-skipping strategy. If inequation (15) is satisfied, indicating that the implementation of the stop-skipping strategy can reduce the route cost, then it should be implemented. The set of bus routes needs to be adjusted by the stop-skipping strategy is denoted by $L_{1}$.

#### Truncation strategy

The truncation strategy refers to skipping the bus stops not only in the affected section but also in the upstream section (or the downstream section), that is, the entire bus route only retains the downstream section (or the upstream section). For any bus route, if the truncation strategy can reduce the route cost, then the truncation strategy is adopted. Notice that if a bus route meets the criteria of both the stop-skipping and the truncation strategies at the same time, then the truncation strategy is adopted.

The total travel time of passengers and the total operating cost of bus route $l \in L$ is shown in “Stop-skipping strategy” (see Eqs. (2) and (7)). With the truncation strategy, the total travel time of passengers on bus route $l \in L$ is as follows:

16 $$T_{2,l} = \sum\limits_{{(i,j) \in A_{l} }} {\left[ {v_{ij,l}^{t} \cdot (t_{ij}^{c} )} \right]} + \sum\limits_{{(i,j) \in D_{l} }} {[v_{ij,l}^{t} \cdot (t_{ij}^{c} + d_{i,l} )]} ,$$

where $v_{ij,l}^{t}$ is the flow of link $(i,j)$ on bus route $l \in L$ after the implementing of truncation strategy and is given by:

17 $$v_{ij,l}^{t} = \sum\limits_{r \le i} {\sum\limits_{s \ge j} {q_{rs,l}^{tru} } } ,$$

where $q_{rs,l}^{tru}$ is the traffic demand from bus stop $r$ to $s$ on bus route $l \in L$ after implementing the stop-skipping strategy and is given by

18 $$q_{rs,l}^{{q_{rs,l}^{tru} }} = \left\{ {\begin{array}{*{20}c} {0,} & {if \, r/s \in A_{l} \cup D_{l} } \\ {q_{rs,l} ,} & {else.} \\ \end{array} } \right.$$

With the truncation strategy, the total operating cost of bus route $l \in L$ under construction is as follows:

19 $$OC_{2,l} = c_{0} \cdot T_{l}^{t} ,$$

where $T_{l}^{t}$ is the roundtrip time of bus route $l \in L$ during construction after implementing the truncation strategy, which is given by

20 $$T_{l}^{t} = \sum\limits_{{(i,j) \in U_{l} }} {(t_{ij}^{c} + d_{i} )} .$$

The passenger loss caused by the truncation strategy is:

21 $$P_{2,l} = \gamma \cdot \sum\limits_{r} {\sum\limits_{s} {q_{rs,l} } , \, r/s \in A_{l} \cup U_{l} } .$$

The criterion for implementing the stop-skipping strategy $c_{2}$ is given by:

22 $$(\lambda \cdot T_{2,l} + OC_{2,l} + P_{2,l} ) - (\lambda \cdot T_{0,l} + OC_{0,l} ) < 0.$$

The two terms on the left-hand side of inequation (22) represent the route cost with and without the truncation strategy. If inequation (22) is satisfied, indicating that the implementation of the truncation strategy can reduce the route cost, then it should be implemented. The set of bus routes that need to be adjusted by the truncation strategy is denoted by $L_{2}$. If $l \in L_{1} \cap L_{2}$, then we adopt the truncation strategy. The bus routes significantly affected by construction but do not meet the criteria of stop-skipping and truncation strategies are denoted by $L_{3}$, and $L_{3} = \left\{ {l, \, l \in \overline{L} \backslash (L_{1} \cup L_{2} ))} \right\}$.

### Step III: optimal design for feeder bus service

The aforementioned two strategies may result in the reduction of service coverage since some previous stops are not served. In Step III, the feeder bus service is designed to guarantee spatial coverage nearby the construction area on the one hand. On the other hand, for bus routes $l \in L_{3}$, the feeder bus service is also designed to replace bus services in the affected sections and to connect the upstream and downstream sections. The upstream and downstream sections of each route are operated independently according to the original bus stops and frequency. Passengers across sections require transfers between the upstream/downstream section and the feeder bus route.

Let $A$ denote the set of bus stops that need to be served by the feeder bus. Then $A$ consists of the skipped stops on bus routes $l \in L_{1} \cup L_{2}$ and the stops in the affected sections of bus routes $l \in L_{3}$.The feeder bus needs to meet passenger demand related to these stops, including passengers within the affected sections and passengers across different sections, which is given by

23 $$q_{ij}^{f} = \sum\limits_{{l \in L_{3} }} {q_{ij,l} } + \sum\limits_{{l \in L_{3} }} {\sum\limits_{{i \in U_{l} }} {q_{ij,l} } } .$$

The starting and terminal stops of the feeder bus service are the first and last stops in set $A$, which are represented by $o$ and $d$, respectively. As the travel time of the links under construction becomes longer, not only the original affected stops but also the stops nearby the affected stops can be alternative stops of the feeder bus route. Let $A^{\prime}$ denote the set of alternative stops of the feeder bus route. Bus companies generally aim to reduce operating costs and passenger travel costs. The objective of the feeder bus service in this paper is to minimize the total system cost $(TSC)$, including the operating cost $(OC)$ and the total travel cost $(TTC)$. Then we build the following model for the optimal design of the feeder bus service:

24 $$\begin{aligned} \min \,TSC & = OC + TTC \\ & = f \cdot c_{0} \cdot (\sum\limits_{i} {\sum\limits_{j} {t_{ij}^{c} \cdot x_{ij} } } ) + \lambda \cdot \sum\limits_{i} {\sum\limits_{j} {(t_{ij}^{c} + d)x_{ij} v_{ij} } } + \gamma \cdot \frac{{\sum\limits_{r} {\sum\limits_{s} {q_{rs}^{new} - \widehat{q}} } }}{2 \cdot f}, \\ \end{aligned}$$

subject to

25 $$\sum\limits_{{j \in A^{\prime}}} {x_{oj} } = 1,$$

26 $$\sum\limits_{{j \in A^{\prime}}} {x_{jo} } = 0,$$

27 $$\sum\limits_{{i \in A^{\prime}}} {x_{id} } = 1,$$

28 $$\sum\limits_{{i \in A^{\prime}}} {x_{di} } = 0,$$

29 $$\sum\limits_{{i \in A^{\prime}}} {x_{ij} } - \sum\limits_{{i \in A^{\prime}}} {x_{ji} } = 0, \, \forall j \in A^{\prime},$$

30 $$\sum\limits_{i,i \ne j} {x_{ij} } \le 1, \, \forall j \in A^{\prime},$$

31 $$\sum\limits_{j,j \ne i} {x_{ij} } \le 1, \, \forall i \in A^{\prime},$$

32 $$x_{ij} + x_{ji} \le 1, \, \forall i \in A^{\prime}, \, i \ne j,$$

33 $$v_{ij} \le (\sum\limits_{i} {\sum\limits_{j} {q_{ji}^{f} )} } \cdot x_{ij} , \, \forall i,j \in A^{\prime},$$

34 $$\sum\limits_{{i \in A^{\prime}}} {x_{ij} } = 1, \, \forall j \in A\backslash o,$$

35 $$\sum\limits_{{j \in A^{\prime}}} {x_{ij} } = 1, \, \forall i \in A\backslash d,$$

36 $$\sum\limits_{i} {x_{ij} \cdot v_{ij} } + \sum\limits_{i} {q_{ji}^{f} } = \sum\limits_{i} {x_{ji} \cdot v_{ji} } ,\forall j,$$

37 $$\frac{{v_{ij} }}{f \cdot Cap} \le LF_{\max } , \, \forall i,j \in A^{\prime},$$

38 $$x_{ij} = \left\{ {0,1} \right\}, \, \forall i,j \in A^{\prime}, \, i \ne j,$$

where decision variables $x_{ij}$ are binary variables; $x_{ij} = 1$ if the feeder route passes through node $j$ immediately after node $i$; $x_{ij} = 0$, otherwise. Decision variables $v_{ij}$ are continuous variables, which represent the link flow on link $(i,j)$. Equation (24) defines the objective function. The first term represents the operating cost, where $c_{0}$ is a constant parameter for the unit operating cost per vehicle hour. $t_{ij}^{c}$ is the bus travel time of link $(i,j)$ on bus route $l$ during construction. The second term represents passenger travel cost, including the inter-stop travel time and the dwell time. The third term denotes the penalty for losing bus passengers, where $\gamma$ is the penalty for losing one passenger. Constraints (25)–(28) define the starting stop and the terminal stop of the feeder bus route. Constraint (29) ensures that each node on the route has one preceding and one following node. Constraints (30) and (31) ensure that each node can be visited at most once. Constraint (32) ensures that the feeder bus route is one-way. Constraint (33) ensures that $v_{ij}$ can take a positive value only if link $ij$ is connected, i.e., $x_{ij} = 1$, which is formulated by the “big-M” method. Constraints (34)–(35) ensure that each origin or destination can be served by the feeder bus route once. Constraint (36) depicts the flow conservation condition. Constraint (37) ensures that the link flow would not exceed the capacity, where $Cap$ is the vehicle capacity, $LF_{\max }$ is the maximum load factor. Constraint (38) defines the binary variable.

The model defined by Eqs. (24)–(38) includes binary variables and continuous variables. It is a MILP model, which can be conveniently solved by some off-the-shelf solvers, such as Gurobi.

## Case study

### Study network and parameter values

**Figure 3:**
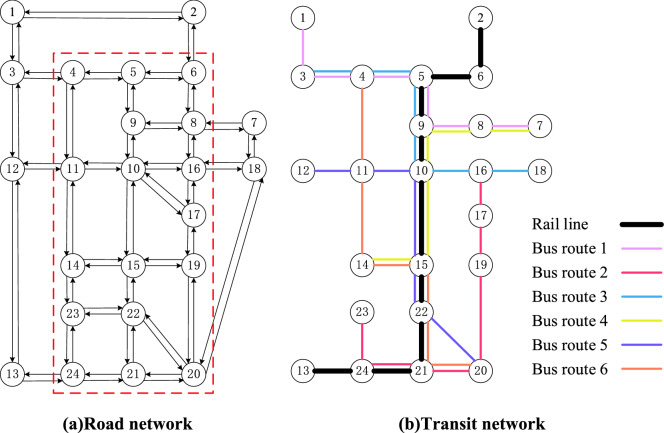
Sioux-Falls road and transit networks.

To demonstrate the effectiveness of the proposed adjustment framework, a case study is conducted in this section. A hypothetical transit network based on the Sioux-Falls network (Fig. 3) is used to illustrate the proposed methods. Table 2 shows the initial transit line data consisting of a rail transit line and six bus routes, including bus frequency, transit stop sequence, and the inter-stop travel time information. Particularly, travel times of affected links before and during construction are given in Table 3; travel times during construction are generated randomly within a certain range. The OD demand data are randomly generated. In this study, we use the Gurobi solver under default settings to solve the proposed model. The following values of parameters are used: $R_{0} = 10\%$, $c_{0} = 20(\$ /veh \cdot {\text{h}})$, $\lambda = 15(\$ /{\text{h}})$, $\gamma = 9$, $t_{a} = 2({\text{s}})$, $t_{b} = 1.5({\text{s}})$.

**Table 2:** Initial transit line data.

Line ID	Frequency	Stop sequence	Inter-stop travel time
Rail transit line	–	2-6-5-9-10-15-22-21-24-13	–
Bus route 1	10	1-3-4-5-9-8-7	4-4-2-5-10-3
Bus route 2	10	16-17-19-20-21-24-23	2-2-4-6-3-2
Bus route 3	10	3-4-5-9-10-16-18	4-2-5-3-4-3
Bus route 4	10	7-8-9-10-15-14	3-10-3-6-5
Bus route 5	10	12-11-10-15-22-20	6-5-6-3-5
Bus route 6	10	4-11-14-15-22-21-20	6-4-5-3-2-6

**Table 3:** Link travel time before and during construction.

Affected bus link	(5, 9)	(9, 10)	(10, 15)	(15, 22)	(22, 21)	(21, 24)
Travel time before construction (min)	5	3	6	3	2	3
Travel time during construction (min)	9	5	12	5	5	6

### Results

According to Eq. (1), the increasing rate of roundtrip time of all bus routes is calculated. The values are 13.3%, 14.4%, 25.8%, 27.7%, 29.8%, and 17.6%, respectively. Since $R_{l}$ for all bus routes are greater than $R_{0} \, (10\% )$, so all bus routes are significantly affected by rail construction and need adjusting. Based on Eqs. (15) and (22), the stop-skipping strategy should be implemented for bus route 1, and the truncation strategy should be implemented for bus route 2. This result is consistent with intuitive understanding. For bus route 1, the affected section is short and the downstream section is long. The travel time saved by the stop-skipping strategy for passengers in the downstream section is greater than its impact on the passengers in the affected section. Therefore, the stop-skipping strategy is effective and should be executed. For bus route 2, the affected section and the downstream section are both short. There are very few passengers involved in the two sections. The benefit of transporting these passengers is less than the operation cost for buses to run through the affected section and the downstream section. Therefore, it is not cost-effective to pass the section under construction and the truncation strategy should be executed.

The values of $R_{l}$ for routes 1 and 2 after adjustment are 9.6% and − 24.0%, respectively. The negative sign indicates that the roundtrip time after implementing the adjustment strategies is less than that before rail construction. It is intuitive since there is one less link for route 2 after implementing the truncation strategy, so the roundtrip time is greatly reduced. For the remaining bus routes, $R_{l}$ is no longer calculated because the routes have been completely changed and redesigned as feeder bus service.

**Figure 4:**
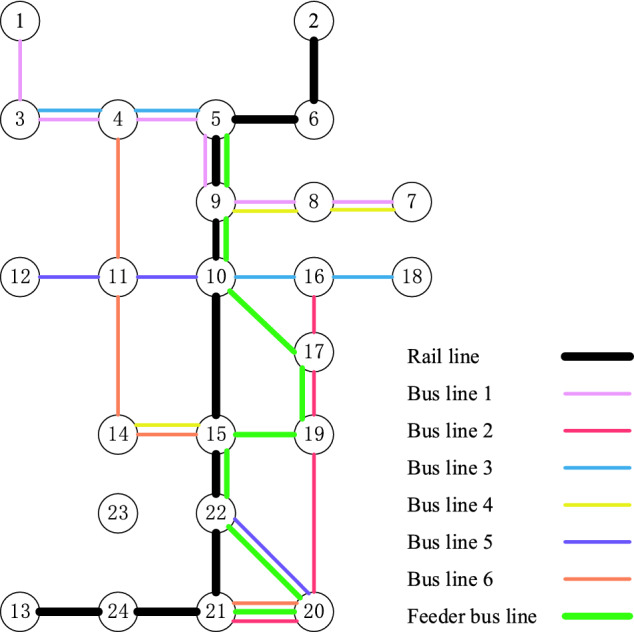
Optimal route configuration of the feeder bus.

Feeder bus service is designed to optimize bus routes 3, 4, 5, and 6. The starting and terminal stops of the feeder bus are stops 5 and 21, respectively. The set of affected stops of the four bus routes is given by $A = \left\{ {5,9,10,15,22,21} \right\}$. The alternative stops in this case are all stops within the red dotted route in Fig. 3a. By solving the model defined by Eqs. (24)–(38), the optimal stop sequence of the feeder bus route is 5-9-10-17-19-15-22-20-21, as shown in Fig. 4. The entire bus route adjustment schemes are shown in Table 4.

**Table 4:** Route adjustment schemes.

Original bus route	Adjustment strategy	Bus route after adjustment
1-3-4-5-9-8-7	The stop-skipping strategy	1-3-4-(5-9)-8-7
16-17-19-20-21-24-23	The truncation strategy	16-17-19-20-21
3-4-5-9-10-16-18	Designing feeder bus	3-4-5, 10-16-18
7-8-9-10-15-14	Designing feeder bus	7-8-9, 15-14
12-11-10-15-22-20	Designing feeder bus	12-11-10, 22-20
4-11-14-15-22-21-20	Designing feeder bus	4-11-14-15, 21-20

The numbers in parentheses indicate the bus stops that are skipped.

An evaluation of the proposed approach is employed by calculating the total cost before and after the route adjustment scheme. The variation in the number of passengers and route costs with the route adjustment scheme is shown in Table 5. The results show that the design of the stop-skipping strategy, the truncation strategy, and the feeder bus service reduces the total cost of the corresponding bus routes by 3.8%, 5.9%, and 13.6%, respectively. The entire adjustment framework reduces the total cost of all bus routes by 9.2%, which demonstrates the effectiveness of the proposed approach.

**Table 5:** The variation of the number of passengers and route costs.

Bus route	Route 1	Route 2	Route 3–6 and the feeder bus	Total
The number of passengers	− 21.7%	− 32.2%	0	− 3.7%
Route costs	− 3.8%	− 5.9%	− 13.6%	− 9.2%

To evaluate the impact of the adjustment scheme on traffic congestion, we define bus vehicle time $(BVT)$ as the product of bus frequency and the travel time on the affected section. Then the total bus vehicle time before and after the adjustment scheme is calculated as follows:

39 $$BVT = \sum\limits_{l} {\sum\limits_{(i,j)} {t_{ij}^{c} \cdot f_{l} } } , \, l \in L, \, (i,j) \in A_{l} ,$$

40 $$BVT^{\prime} = \sum\limits_{l} {\sum\limits_{(i,j)} {t_{ij}^{c} \cdot f_{l} } } , \, l \in L_{1} \cup L_{fb} , \, (i,j) \in A_{l} ,$$

where $L_{fb}$ represents the feeder bus service. We calculate the total bus vehicle time passing through the affected section before and after the adjustment scheme. The results indicate that the total bus vehicle time passing through the affected section is reduced by 28.2%. Hence, the adjustment scheme can mitigate traffic congestion resulting from the construction of rail transit.

### Sensitivity analysis

To analyze the impact of increased link travel time caused by rail construction on the route adjustment scheme, an increasing proportion $\theta$ is defined to denote the increased proportion of link travel time under construction. The adjustment schemes with different increased proportions of link travel time are obtained, as shown in Table 6. Other parameters are consistent with “Study network and parameter values”. When $\theta$ is equal to 1.2, $R_{l}$ of all bus routes are smaller than $R_{0} \, (10\% )$, indicating that the influence of rail construction on all the six bus routes is small, and none of them need adjusting. As $\theta$ continues to grow, the number of bus routes that need adjusting grows. For each bus route, the longer the travel time of effected sections, the more pressing it needs adjusting. When $\theta$ equals 1.8, the stop-skipping strategy is needed for route 1 to achieve a smaller route cost. When $\theta$ equals 2.0, the truncation strategy is needed for route 2 to avoid the large operating costs of affected sections.

**Table 6:** Adjustment schemes with different increased proportion of link travel time.

Bus route	$R_{l}$ and adjustment scheme with different $\theta\theta$									
	$\theta { = }1.2$		$\theta { = }1.4$		$\theta { = }1.6$		$\theta { = }1.8$		$\theta { = 2}{\text{.0}}$	
Route 1	3.3%	*N*	6.6%	*N*	10.0%	*N*	13.3%	*S*	16.6%	*S*
Route 2	2.9%	*N*	5.8%	*N*	8.6%	*N*	11.5%	*FB*	14.4%	*T*
Route 3	6.9%	*N*	13.8%	*FB*	20.7%	*FB*	27.6%	*FB*	34.5%	*FB*
Route 4	6.2%	*N*	12.5%	*FB*	18.7%	*FB*	24.9%	*FB*	31.1%	*FB*
Route 5	6.7%	*N*	13.4%	*FB*	20.1%	*FB*	26.8%	*FB*	33.5%	*FB*
Route 6	3.5%	*N*	7.0%	*N*	10.6%	*FB*	14.1%	*FB*	17.6%	*FB*

Hereinafter, adjustment scheme *N* represents the bus route does not need be adjusted. Adjustment schemes *S*, *T*, and *FB* represent the stop-skipping strategy, the truncation strategy, and the feeder bus service should be implemented, respectively.

Sensitivity analysis of $R_{0}$ is intuitional. If $R_{0}$ is greater than 10%, all adjustment schemes for lines satisfies $R_{l} \le R_{0}$ in Table 2 should be changed to *N*. If $R_{0}$ is less than 10%, the adjustment schemes for lines satisfies $R_{0} < R_{l} \le 10\%$ in Table 2 should be changed to *FB*.

To analyze the impact of the penalty for losing one passenger on the route adjustment scheme, a sensitivity analysis of $\gamma$ is conducted. Other parameters are consistent with “Study network and parameter values”. As can be seen from Table 7, as $\gamma$ continues to grow, the stop-skipping strategy and the truncation strategy are no longer appropriate. Because both of them sacrifice the travel of some passengers. When $\gamma = 12$, the feeder bus route includes two more stops compared with the feeder bus route in “Results”, this is because the feeder bus should serve bus sections 21-24-23 skipped by bus route 2, with a high penalty for losing passengers. When $\gamma$ is greater than 15, all of the six bus routes should be optimized by designing feeder bus services.

**Table 7:** Adjustment schemes with different penalties for losing one passenger.

Bus route	1	2	3	4	5	6	Feeder bus configuration
$\gamma = 3$	*S*	*T*	*FB*	*FB*	*FB*	*FB*	5-9-10-17-19-15-22-20-21
$\gamma = 6$	*S*	*T*	*FB*	*FB*	*FB*	*FB*	5-9-10-17-19-15-22-20-21
$\gamma = 9$	*S*	*T*	*FB*	*FB*	*FB*	*FB*	5-9-10-17-19-15-22-20-21
$\gamma = 12$	*S*	*T*	*FB*	*FB*	*FB*	*FB*	5-9-10-17-19-15-22-20-21-24-23
$\gamma = 15$	*FB*	*FB*	*FB*	*FB*	*FB*	*FB*	5-9-10-17-19-15-22-20-21

Adjustment schemes *S*, *T*, and *FB* represent the stop-skipping strategy, the truncation strategy, and the feeder bus service should be implemented, respectively.

To analyze the impact of passengers’ value of time on the route adjustment scheme, a sensitivity analysis of $\lambda$ is conducted, as shown in Table 8. When $\lambda$ is small, all of the six bus routes need designing feeder bus services instead of implementing the stop-skipping strategy or the truncation strategy. This is because the penalties for losing passengers outweigh the benefits of saving travel cost for other passengers, with a small passengers’ value of time. As $\lambda$ continues to grow, the stop-skipping strategy and the truncation strategy are more appropriate. When $\gamma = 12$, even all the bus routes should be truncated in order to achieve lower operating costs and passenger travel costs. This is obviously unreasonable in reality. Therefore, when adjusting bus routes, multiple parameters need to be reasonably valued at the same time to ensure the applicability of the scheme in reality. For case study in this paper, the result in “Results” is an applicable scheme.

**Table 8:** Adjustment schemes with different passengers’ values of time.

Bus route	1	2	3	4	5	6	Feeder bus configuration
$\lambda = 5$	*FB*	*FB*	*FB*	*FB*	*FB*	*FB*	5-9-10-17-19-15-22-20-21
$\lambda = 10$	*S*	*T*	*FB*	*FB*	*FB*	*FB*	5-9-10-17-19-15-22-20-21
$\lambda = 15$	*S*	*T*	*FB*	*FB*	*FB*	*FB*	5-9-10-17-19-15-22-20-21
$\lambda = 20$	*T*	*T*	*FB*	*T*	*T*	*T*	5-9-10-17-19-15-22-20-21-24
$\lambda = 25$	*T*	*T*	*T*	*T*	*T*	*T*	–

Adjustment schemes *S*, *T*, and *FB* represent the stop-skipping strategy, the truncation strategy, and the feeder bus service should be implemented, respectively.

## Conclusions

This paper proposed a three-step adjustment framework to actively mitigate the impact of rail transit construction on bus operations. In Step I, a criterion was developed for selecting bus routes which are significantly affected due to rail transit construction and necessitate being adjusted. In Step II, we designed the stop-skipping strategy and truncation strategy, and checked whether they are opportune to ameliorate the service of each affected bus route. In Step III, feeder bus services were further designed to guarantee spatial coverage nearby the construction area. A mixed-integer linear programming model was later developed to determine the optimal configuration of the feeder bus service.

A case study based on the Sioux-Falls network was conducted to demonstrate the applicability of the proposed framework. The results show that the proposed adjustment framework can reduce the total cost of bus routes $l \in \overline{L}$ by 9.2%; the feeder bus service can reduce the total cost of bus routes $l \in L_{3}$ by 13.6%. The total bus vehicle time passing through the affected section was reduced by 28.2%. Hence, the adjustment scheme can also mitigate traffic congestion resulting from the construction of rail transit.

The method proposed in this paper is not only applicable to the adjustment of bus routes during the construction of rail transit but also can be applied to other circumstances such as road maintenance. The paper could be extended in the following directions. First, the feeder bus service may attract more passengers than the sum of the passengers on the initial lines. On the other hand, the environmental deterioration brought by rail construction may lead to the loss of passengers. The design of the bus adjustment scheme taking into account passengers’ perception of rail construction and the induced passengers by feeder buses is left for a future ongoing study. Secondly, more factors such as the evolution of passenger access/egress trips and the accessibility of bus services, as well as calibration/validation against real data could be taken into account.
